# Brain-inspired global-local learning incorporated with neuromorphic computing

**DOI:** 10.1038/s41467-021-27653-2

**Published:** 2022-01-10

**Authors:** Yujie Wu, Rong Zhao, Jun Zhu, Feng Chen, Mingkun Xu, Guoqi Li, Sen Song, Lei Deng, Guanrui Wang, Hao Zheng, Songchen Ma, Jing Pei, Youhui Zhang, Mingguo Zhao, Luping Shi

**Affiliations:** 1 Department of Precision Instrument, Center for Brain-Inspired Computing Research (CBICR), Beijing Innovation Center for Future Chip, Optical Memory National Engineering Research Center, Tsinghua University, Beijing, China; 2 Department of Computer Science and Technology, Tsinghua University, Beijing, 100084 China; 3 Department of Automation, Tsinghua University, Beijing, 100084 China; 4 Laboratory of Brain and Intelligence, Department of Biomedical Engineering, IDG/ McGovern Institute for Brain Research, CBICR, Tsinghua University, Beijing, China; 5 Lynxi Technologies Co., Ltd, Beijing, China

**Keywords:** Engineering, Mathematics and computing, Computer science

## Abstract

There are two principle approaches for learning in artificial intelligence: error-driven global learning and neuroscience-oriented local learning. Integrating them into one network may provide complementary learning capabilities for versatile learning scenarios. At the same time, neuromorphic computing holds great promise, but still needs plenty of useful algorithms and algorithm-hardware co-designs to fully exploit its advantages. Here, we present a neuromorphic global-local synergic learning model by introducing a brain-inspired meta-learning paradigm and a differentiable spiking model incorporating neuronal dynamics and synaptic plasticity. It can meta-learn local plasticity and receive top-down supervision information for multiscale learning. We demonstrate the advantages of this model in multiple different tasks, including few-shot learning, continual learning, and fault-tolerance learning in neuromorphic vision sensors. It achieves significantly higher performance than single-learning methods. We further implement the model in the Tianjic neuromorphic platform by exploiting algorithm-hardware co-designs and prove that the model can fully utilize neuromorphic many-core architecture to develop hybrid computation paradigm.

## Abstract (web summary)

Global and local learning represent two distinct approaches to artificial intelligence. In this manuscript, Wu et al present a hybrid learning strategy, drawing from elements of both approaches, and implement it on a co-designed neuromorphic platform.

## Introduction

The majority of neuromorphic models are established by single backpropagation or single neuroscience-oriented local plasticity (LP), exhibiting radicals with different learning features and advantages. In general, backpropagation is global error-driven learning with two alternative information circuits (top-down and bottom-up). The learning process is implemented via layer-by-layer allocation of global supervised errors. Benefitting from the recent progress on deep learning, backpropagation and its many variants have been applied for training neuromorphic models represented by spiking neural networks (SNNs) and demonstrated good accuracy in certain specific tasks, such as image classification and reinforcement learning (RL)^1–8^. In contrast, neuroscience-oriented LP is essentially correlation-driven learning, which prominently occurs between presynaptic and postsynaptic neurons and is triggered by asynchronous spike activity. Rooted in neuroscience, LP has been widely used for feature abstraction and facilitates the realization of many advanced brain-inspired learning and memory mechanisms^9–12^. Despite remarkable practical advantages in low-latency and energy-efficiency computation^13,14^, applications of neuromorphic models are still limited to a small range of usages and the overall performance is inferior to the state-of-the-art results^15,16^. To fully exploit the potential still requires many effective algorithms. At the same time, local correlation-driven learning (called LP) and global error-driven learning (called “global plasticity” (GP)) are the two principal learning routes for artificial intelligence (AI). Both approaches have their unique advantages but neither can completely outperform the other on all learning problems. Thus, it is highly expected to integrate them into one single neuromorphic hybrid model to explore complementary synergic learning capabilities in multiple learning scenarios. However, the different learning circuits and weight update behaviors make the development of such hybrid learning obscure and the further incorporation of complex and diverse spiking dynamics poses a greater challenge for exploring hybrid learning on neuromorphic models.

Recently, there has been an increasing number of studies involving global-local hybrid models with different interests. A related biological study was on the three-factor learning rule^17,18^. The three-factor learning rule describes a general framework of synaptic plasticity that incorporates presynaptic activity, postsynaptic activity, and additional third factors, such as neuromodulators and neurotransmitters, representing the top-down supervised signals. Several studies on developing spiking learning algorithms have been inspired by the three-factor learning rule^8,19–23^. Adopting a biologically plausible manner, they took supervised errors or rewards as the biological third factor to modulate the magnitude of local weight update and successfully applied the methods for simple image classification tasks^8,19^, RL tasks^20^ and probabilistic inference tasks^21–23^. Despite great progress in understanding biological learning, due to the different learning goals, most of the works fail to fully exploit the advantages of global gradient learning and are generally not good at solving many complex learning problems^24,25^.

Another related vein is meta-learning. Meta-learning, also named learning-to-learn, is ubiquitous in nature for continuously improving the learning ability. Existing meta-learning studies^26–28^ for SNNs narrowly focus on improving a single GP-based model without integrating LP. How to learn to optimize both the LP and GP with various spiking features and integrate their respective advantages in one neuromorphic model remains an important open issue. Alternatively, in the early 1990s, ref. ^29^ proposed a framework that can optimize LP through supervised signals. In follow-up research, several studies extended the framework to establish large-scale non-SNNs and have demonstrated high performance in solving few-shot learning^30,31^ and unsupervised learning^32,33^. However, due to the lack of an effective and configurable hybrid mechanism, the potential of global-local learning has not yet been fully explored, either in some important learning capabilities or computational efficiency. Furthermore, none of the works considered developing such hybrid synergic learning in neuromorphic computing.

The previous lack of powerful hybrid learning for neuromorphic models also affects the use of many-core neuromorphic hardware, which aims to provide ultralow-power hardware solutions for AI implementations. A feasible hardware scheme that can support online hybrid learning has yet to be reported. If neuromorphic hybrid learning models with algorithm-hardware co-design could be developed on neuromorphic platforms, then the neuromorphic many-core architecture can be exploited to explore hybrid on-chip computation schemes to obtain better performances in practical learning scenarios.

Here, we show a spike-based hybrid plasticity (HP) model using a brain-inspired meta-learning paradigm and a differential spiking dynamics model with parameterized LP. The approach provides a generic and flexible integration of these two learning methods and facilitates high-efficiency hybrid learning on neuromorphic chips. By developing multiple synergic learning strategies, we demonstrate that, with slight modifications to the local modules, the proposed model can solve three different learning problems, including few-shot learning, continual learning, and fault-tolerance learning. Finally, we exploit the method of the algorithm-hardware co-designs to implement the hybrid model on Tianjic chips and further prove that it can fully utilize neuromorphic architecture to develop a hybrid computation paradigm.

## Results

### Hybrid synergic learning model

**Fig. 1:**
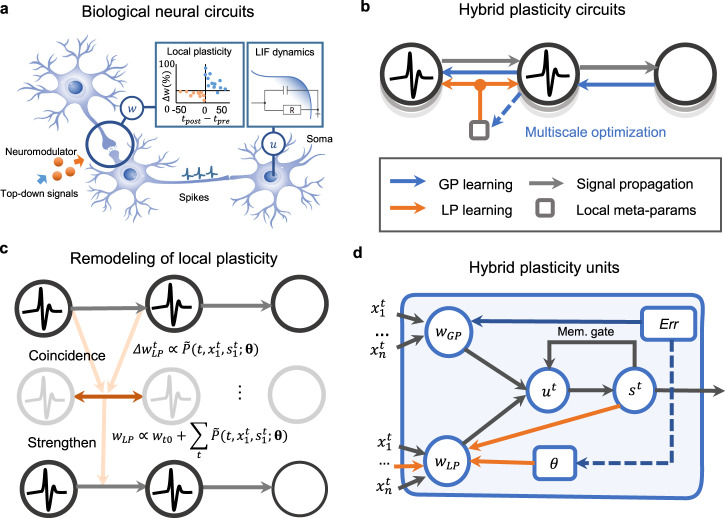
Illustration of hybrid synergic learning model. **a** An illustration of biological synaptic plasticity mechanisms and neuronal dynamics. The neuromodulators exhibit a radically different evolutionary (learning) process from synaptic plasticity and can encode top-down supervision information to modulate versatile synaptic plasticity behaviors. **b** Motivated by the neural circuitry, we develop a multiscale meta-learning paradigm to integrate these two types of learning in one neuromorphic model. It models the parameters of local learning as a type of meta-learning parameters and decouples the learning process of weights (solid blue lines) and these local meta-parameters (dashed blue lines) by using a bilevel optimization technique, enabling flexible multiscale learning. **c** Parametric biological short-term plasticity. Local weight modifications can be equivalently modeled as a parametric function related to the initial weight value$\,{w}_{{t}_{0}}$, the presynaptic spike activity ${x}_{1}^{t}$, the postsynaptic spike activity, ${s}_{1}^{t}$, and the hyperparameters ${{{{{\boldsymbol{\theta }}}}}}$ of the Hebbian rule. **d** The HP unit divides the weights into two branches, ${w}_{GP}$ and ${w}_{LP}$. ${w}_{GP}$ is updated based on global errors, and ${w}_{LP}$ is updated based on adjacent neuron activities and meta-parameters. In each HP unit, we use a memory gate that controls the reset or leaky integration behaviors of membrane potential$\,{u}_{t}$ using the spike firing state$\,{s}_{t}$.

The proposed spike-based hybrid model exploits two streams of neuroscience experimental cues about synaptic modulation behaviors and a multiscale learning mechanism (Fig. 1a) to model the neuromodulatory mechanism and establish the synergic learning circuit.

First, in the hippocampus, local neural circuits can be simultaneously controlled by multiple types of top-down modulatory signals. These signals act on many synapses and modulate diverse plasticity behaviors, including the learning rate, update polarity, and plasticity consolidation^17,34,35^. In particular, some neuromodulators, such as adenosine, can affect the actions of synaptic functioning and other modulators in a hierarchical manner^36,37^. This indicates that neuromodulators can be formulized as a special type of meta-learning parameter acting on synaptic plasticity in a weight-sharing manner.

Second, neuromodulators can modulate synaptic plasticity on multiple temporal scales. Neuromodulators exhibit radically different evolution (learning) scales and individual functions from synapses^35,38^. The coupling of plasticity and neuromodulatory mechanisms plays a vital role in building complex behavior functions, such as muscle contraction^39^. It further implies a flexible and multiscale learning mechanism in which the learning process of neuromodulators and synapses may occur on different spatial and temporal scales; thus, they can be formulized as two types of variables with different learning manners in the optimization process.

To this end, we formulize the hyperparameters of LP, such as the learning rate and sliding threshold, as a group of meta-parameters ${{{{{\boldsymbol{\theta }}}}}}$. Since these meta-parameters ${{{{{\boldsymbol{\theta }}}}}}$ control the weight update behaviors, we propose modeling ${{{{{\boldsymbol{\theta }}}}}}$ as the upper-level variable of weight $w$ and transforming the learning process of synaptic weights and neuromodulators into a bilevel optimization problem^40^. On this basis, we decouple the learning process of ${{{{{\boldsymbol{\theta }}}}}}$ and $w$ in different optimization loops, and this allows to use the hyperparameter optimization technique^41^ for optimizing ${{{{{\boldsymbol{\theta }}}}}}$ (see Methods). In this manner, we expect to first provide an effective flexible modeling strategy that supports the modeling of versatile local learning rules and can learn to optimize both the underlying weights and the local learning rules in different optimization loops, thereby facilitating a generic integration of the two types of learning.

Furthermore, we want to incorporate diverse spiking dynamics, LP, and global learning into one unified temporal credit assignment problem. To this end, we jointly derive from membrane potential dynamics and ion-channel dynamics and obtain a differentiable signal propagation equation of subthreshold dynamics. We use a memory gate (Fig. 1d) and make a careful choice of derivative approximation^8,42^ for handling the discontinuous dynamics of spiking neurons (see Methods). Then, we adopt the backpropagation through time (BPTT) algorithm as global learning for training SNNs. Because LP has an independent correlation-based updating manner, directly integrating the local modules with handcrafted parameters and global learning together is difficult to ensure convergence. To incorporate the impact of local weight updates into the entire optimization framework, we take a parametric modeling strategy (Fig. 1c) to transform the local synaptic increments $\Delta {w}_{{LP}}$ into a parametric function related to presynaptic spike activity, *pre*, postsynaptic spike activity, *post*, and local hyperparameters ${{{{{\boldsymbol{\theta }}}}}}$, consisting of the local learning rate, sliding threshold and some other hyperparameters determined by specific local learning rules. By doing so, we not only maintain the underlying weight directly optimized by BPTT but also exclusively model local weight updated behaviors as a temporal-based function concerning adjacent spiking activities and local meta-parameters.

The adopted decoupling optimization strategy is also inspired by a variant of synaptic dynamics. Specifically, derived from the ion-channel differential dynamics, the synaptic weights $w\left(t\right)$ have two terms

1 $$w\left(t\right)=w\left({t}_{n}\right){e}^{\frac{{t}_{n}-t}{{\tau }_{w}}}+\widetilde{P}\left(t,{pre},{post}{{{{{\rm{;}}}}}}{{{{{\boldsymbol{\theta }}}}}}\right)\;\triangleq\; {w}_{{GP}}+{w}_{{LP}},$$

where $w\left({t}_{n}\right)$ denotes the phase value of the synaptic dynamics at discrete time ${t}_{n}$, $k\left(t\right)={e}^{\frac{{t}_{n}-t}{{\tau }_{w}}}$ denotes the synaptic decay function, and ${\tau }_{w}$ denotes the synaptic constant. Here $\widetilde{P}\left(t,{pre},{post}{{{{{\rm{;}}}}}}{{{{{\boldsymbol{\theta }}}}}}\right)$ denotes the generic local modifications. If we further assume the top-down signals to modify the state $w\left({t}_{n}\right)$ at the specific time ${t}_{n}$ when supervision signals are provided, and assume that $\widetilde{P}\left(t,{pre},{post}{{{{{\rm{;}}}}}}{{{{{\boldsymbol{\theta }}}}}}\right)$ represents the LP driven by neuron activities and meta-parameters ${{{{{\boldsymbol{\theta }}}}}}$, then Eq. (1) can be used to accordingly decompose the weight into two parts, ${w}_{GP}$ and ${w}_{LP}$.

Finally, we develop a global-local synergic learning model that integrates LP and GP into one neuromorphic model by exclusively allocating them to act on different weight parts and time scales. Here, each HP unit has two weight branches (Fig. 1d): ${w}_{GP}$, being directly updated by BPTT when receiving supervision information, and ${w}_{LP}$, being updated in an event-driven manner by the meta-learned spiking LP. To fully utilize various learning features of the LP and GP, we use the meta-learning circuit and bilevel optimization technique to decouple the optimization process of ${w}_{LP}$ and ${w}_{GP}.$ The HP model provides a flexible and configurable solution that allows the construction of different learning modules, support various local learning rules, and configures different learning strategies for solving a variety of complex tasks. In particular, the property is quite fascinating for low-energy and efficient execution in edge computing^43^ by reusing limited memory resources and adapting different learning strategies.

### Baseline performance evaluation

We comprehensively evaluated the basic performance, including the accuracy, coding efficiency, and convergence, of the HP models using image classification datasets (including MNIST, Fashion-MNIST, and CIFAR10) and neuromorphic datasets (including CIFAR10-DVS and DVS-gesture). The network architecture and training details are described in Methods.

We analyzed the accuracy of the models on different types of datasets in Table 1. Because SNNs achieve a balance between performance and efficiency, we mainly compared the proposed model with other advanced spike-based models. In image classification tasks, the HP SNN achieves higher accuracies compared with other published work. In sequential learning tasks, the HP SNN improves the performance of a single GP SNN, indicating that local modules are beneficial for the use of longer learning time scales. We provide a deeper analysis in Fig. 3 thereon.

**Table 1:** Comparison of the state-of-the-art results of spike-based networks on several image classification tasks and sequential-based classification tasks.

Model	Dataset	Method	Avg. latency^a^	Accuracy (%)
Spiking MLP^9^	MNIST	LP	350	95.00
Spiking MLP^1^	MNIST	GP	–	97.55
Spiking CNN^2^	MNIST	GP converted^b^	200	99.12
Spiking CNN (this work)	MNIST	${{{{{\rm{HP}}}}}}$	7.5	99.50 ± 0.04
Spiking MLP^3^	F-MNIST	GP based	400	90.13
Spiking CNN (this work)	F-MNIST	${{{{{\rm{HP}}}}}}$	3.9	93.29 ± 0.07
Spiking CNN^63^	CIFAR10	GP converted^b^	500	91.55
Spiking CNN^4^	CIFAR10	GP converted^b^	5	91.78
Spiking CNN (this work)	CIFAR10	${{{{{\rm{HP}}}}}}$	4.5	91.08 ± 0.09
Spiking CNN^59^	CIFAR10-DVS	GP	50	60.05
Spiking CNN (this work)	CIFAR10-DVS	GP	50	67.22 ± 0.43
Spiking CNN (this work)	CIFAR10-DVS	${{{{{\rm{HP}}}}}}$	50	67.81 ± 0.34
Spiking CNN^5^	DVS-Gesture	GP	400 ms	94.13
Spiking CNN^62^	DVS-Gesture	GP	–	96.53
Spiking CNN^6^	DVS-Gesture	GP	400 ms	96.78
Spiking CNN (this work)	DVS-Gesture	GP	400 ms	$96.21\pm 0.32$
Spiking CNN (this work)	DVS-Gesture	${{{{{\rm{HP}}}}}}$	400 ms	$97.01\pm 0.21$

The results of the proposed models in this work collected from ten repeated runs and $\pm$ indicates the 95% confidence interval.

^a^Results from the training time steps or windows reported in the published work.

^b^Converted from a pre-trained GP-based ANN.

**Fig. 2:**
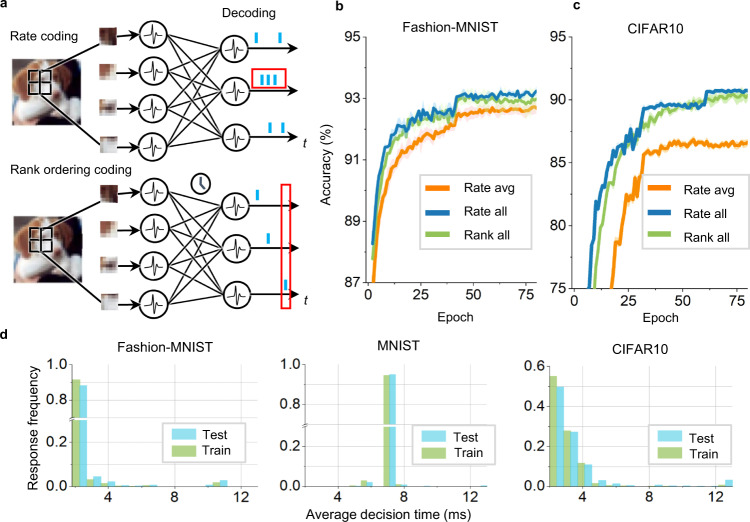
The hybrid plasticity spiking neural network can flexibly support different coding schemes and achieve a trade-off between performance and efficiency. **a** Illustration of rate coding and temporal rank order coding. In rate coding, the network counts the number of spikes fired by the output neurons as the output representation. In temporal rank order coding, the firing time of each neuron is regarded as an encoding of additional information, and the network determines the output in a winner-take-all manner where the firing order of the output neurons is used to output classification results. **b**, **c** Comparison of the training curves of the model with different coding schemes (called Rate-all or Rank-all) for the Fashion-MNIST (**b**) and CIFAR10 (**c**). The average and standard derivations over ten repeated trials are plotted. The average firing time over ranking coding schemes is counted and rounded up, and the rate-based model is tested with this average time window (called Rate-avg.). **d** Average response time for each testing category. We recorded the average response time steps required to produce results for each testing sample when running SNNs with rank order coding in an event-driven inference mode. After that, we counted these time steps and generated a normalized frequency histogram.

One prominent feature of our work is the modeling of synaptic ion-channel dynamics, thereby finding an interesting relationship between spiking information coding and an event-driven inference capability (see Methods). Next, we show that HP SNNs can support rate coding and temporal rank coding schemes^44^ (Fig. 2a). Figure 2b, c compares the results of different coding methods on Fashion-MNIST and CIFAR10. Specifically, we compared the training curves of the SNNs with rank order coding and those with rate coding using the same simulation time windows. Because of the event-driven property of rank order coding, we computed its average time windows and further compared the training curves of the SNN with rate coding using the same average time windows (called rate-avg.) in Fig. 2b, c. These figures indicate that HP SNNs are well suited for rate-based and rank-based spiking networks. With a longer average time window, the rate-based model obtains higher accuracy. With more flexible and event-driven response characteristics, the rank-based model achieves lower inference latency with a slight accuracy loss. Furthermore, we also plot the details of the average response time of each category for different datasets in Fig. 2d. We found that HP SNNs use a flexible strategy for decision-making. Using the CIFAR10 dataset as an example, for most categories, HP SNNs can make decisions within four time windows; however, for some complicated patterns, the model requires more time to make decisions. This flexible decision process significantly reduces the average inference latency and leverages the high efficiency of neuromorphic hardware. We instantiated our model on Tianjic chips and reported the energy evaluation in Supplementary Table 1.

**Fig. 3:**
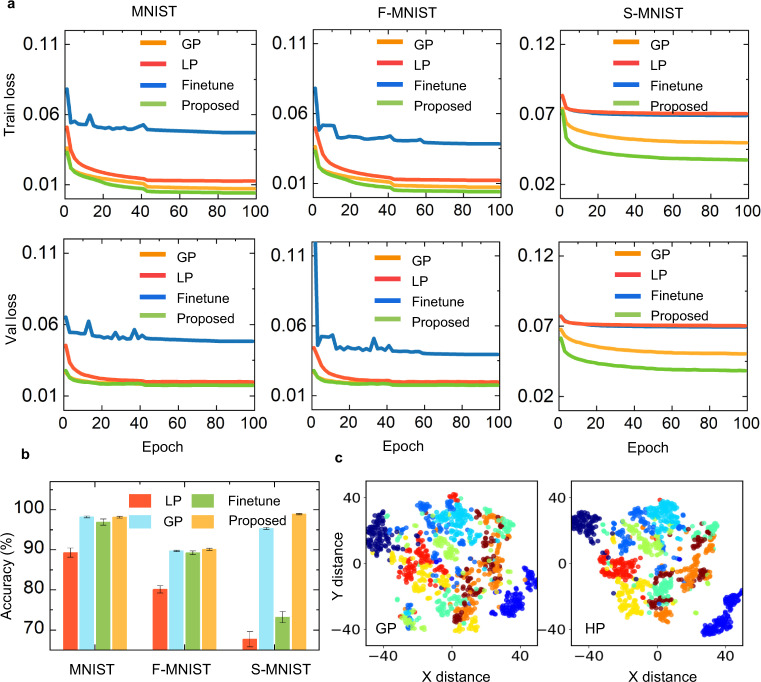
The hybrid plasticity approach can effectively ensure the convergence and accuracy of the proposed hybrid synergic learning. **a** Comparison of the convergence curves of the single GP-based, LP-based, fine-tuning-based (denoted by Finetune) and the proposed HP model over MNIST, Fashion-MNIST (F-MNIST), and Sequential MNIST (S-MNIST). **b** Accuracy histogram for the four models. The error bar indicates the standard derivation for ten repeated trials. *P < 0.05. Two-sided *t*-test was applied to assess statistical significance. **c** T-distributed stochastic neighbor embedding visualization of the average firing rate in the first hidden layer using S-MNIST.

We further compared the convergence of the proposed approach with other related learning methods. Intuitively, since local learning and global learning have independent update methods, directly combining them cannot ensure convergence. Furthermore, regarding neuroscience-oriented learning models, such as STDP-based SNNs, systematically and generally configuring parameters over local learning rules has yet to be resolved. Although hand-designed or biosimulated hyperparameters can alleviate the above problems, it is time-consuming and difficult to ensure performance. By the parametrization of the local module, the HP approach can automatically optimize the local hyperparameters and achieve a synergic learning mode. As a demonstration, we comprehensively compared the loss curves and accuracies of a single LP network, a single GP network, a fine-tuning LP±GP network, and the proposed HP network in Fig. 3. For fairness, we used the same initial weight configurations (see Methods).

Figure 3a shows that the fine-tuning method has poor convergence and performs worse than the other models. Through the proposed compatible design, the HP model significantly improves the accuracy of the LP model (Fig. 3b) and fine-tuning hybrid model, indicating that the proposed method can efficiently integrate the LP and GP methods. The single GP model is suitable for optimizing the errors of common classification tasks. The HP model inherits this advantage and achieves comparable convergence on the static MNIST and Fashion-MNIST datasets. Furthermore, the HP model demonstrates higher accuracy and faster convergence than that of the GP model on the sequential MNIST dataset. Because the LP module provides a correlation-driven weight matrix that stores the correlation product of the presynaptic inputs and postsynaptic activations (i.e., spikes) in the previous steps, as indicated in ref. ^45^, it can act as a type of attention mechanism to the recent past with the strength being determined by the scalar product between the current hidden vector and earlier input stimulus. Combining the results of Table 1 and Fig. 3 shows that adding LP modules could benefit the use of longer learning time scales. Furthermore, we visualized the activations in the first hidden layer by 2D embedding visualization of T-distributed stochastic neighbor embedding over the Sequential MNIST. Take the yellow cluster in Fig. 3c as an example. Adding LP modules can help the HP model abstract the points within each class more compactly and push different classes farther. Overall, the above results indicate that the HP can efficiently coordinate the GP and LP methods with stable convergence for common classification tasks.

### Fault-tolerance learning

**Fig. 4:**
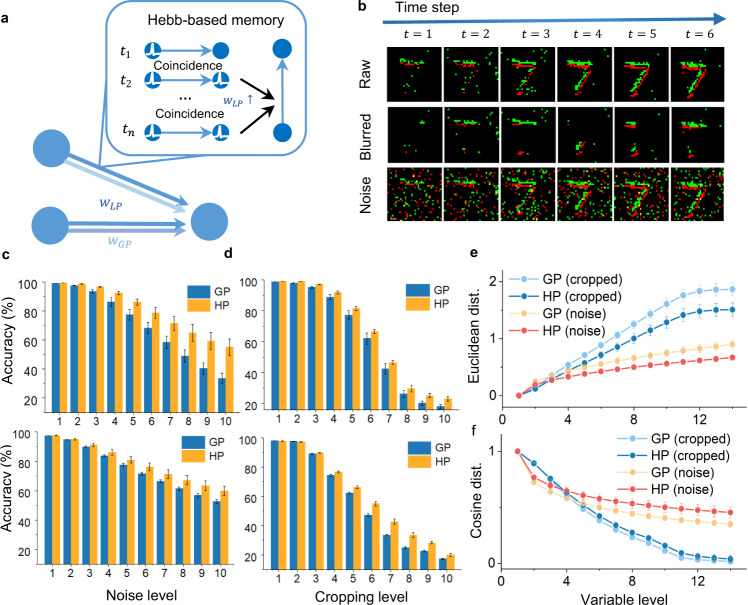
Hybrid plasticity improves fault tolerance. **a** An illustration of the memory function provided by the correlation-based local module. When detecting a strong correlation between presynaptic and postsynaptic neurons, local weight ${w}_{LP}$ consolidates these synaptic weights with a learnable writing and decaying mechanism, which can help memorize repeated patterns and facilitate the recognition of similar stimulus. **b** Generation of incomplete data by cropping (the second row) and noise mixing (the third row) using N-MNIST data. We randomly cropped raw data (the first row) with different cropping sizes or added different levels of salt-and-pepper noise. **c** Performance comparison in the cropping experiments using the N-MNIST (upper) and MNIST (lower). **d** Performance comparison in the noise experiments using the N-MNIST (upper) and MNIST (lower). **e** Euclidean distance between the hidden activation (membrane potential in the last timestep) of cropped MNIST data and those of the original MNIST data. **f** Cosine distance between the hidden activation of cropped MNIST data and original MNIST data. We plotted the average and standard deviations of ten repeated trials in **c**–**f** with *P < 0.05.

Fault tolerance is essential for the real-time information processing of neuromorphic chips to prevent the influence of internal noise or external interference. For example, neuromorphic vision sensors (NVSs)^46^ can quickly capture per-pixel brightness changes with low latency and a high dynamic range but suffer from the inherent noise of physical devices and the movements of external background objects, thereby affecting their practical performance. Next, we demonstrate that by virtue of Hebbian-based local modules (Fig. 4a) and the hybrid strategy, the HP model may improve the fault tolerance of single GP-based networks. We examined the ability of this model to handle incomplete data using an image classification dataset (MNIST) and a neuromorphic dataset (N-MNIST). We used incomplete data to refer to cropping data (e.g., parts of image information are masked, Fig. 4b) and noise-mixed data (data mixed with salt-and-pepper noise, Fig. 4b). The models were trained on the standard datasets and tested using incomplete test samples. Figure 4 shows that as the cropping area increases, the HP model exhibits stronger resistance to the cropping area on the N-MNIST (upper) and MNIST datasets (lower). Furthermore, the noise experiments also show that the HP model achieves good robustness and mitigates the interference of different types of noise. Similarly, the superiority of the HP model becomes apparent as the noise level increases (Fig. 4d). To obtain a more insightful analysis, we calculated the Euclidean distance (Fig. 4e) and the cosine similarity (Fig. 4f) between the first hidden layer activation of the incomplete data and those of the original data using the same model on the MNIST dataset. As Fig. 4f, e illustrates, the HP model diminishes the pattern distance between the incomplete patterns and original patterns, indicating that the local modules can help the network leverage the previous associative features from incomplete data and therefore benefit the network fault tolerance capability. We provide the effectiveness analyses of the HP models in the following section.

### Few-shot learning

We next investigate the potential of HP models for few-shot learning. In this case, the classifier must adapt to new classes not seen in the training phase when only given a limited number of samples from each class. To efficiently establish a mapping relationship from the limited data, it is vital to leverage prior knowledge or acquire inductive biases. The GP-based networks succeed in abstracting useful features; however, it is difficult for the networks to exploit the prior knowledge hidden in the limited datasets without resorting to other techniques. In contrast, the brain is highly efficient in learning from limited data. Neuroscience findings^11^ reveal that the response of cortical neurons to a sensory stimulus can be reliably increased after just a few repetitions by virtue of local synaptic plasticity, indicating that such plasticity may play an important role in exploiting the correlation hidden in limited data. By integrating LP and GP learning, we expect that the synergic learning model can solve this problem through a two-fold mechanism: (1) abstract sufficiently discriminant representation of input data by the GP module; (2) find a useful inductive bias from a limited number of example pairs mainly by the LP module.

**Fig. 5:**
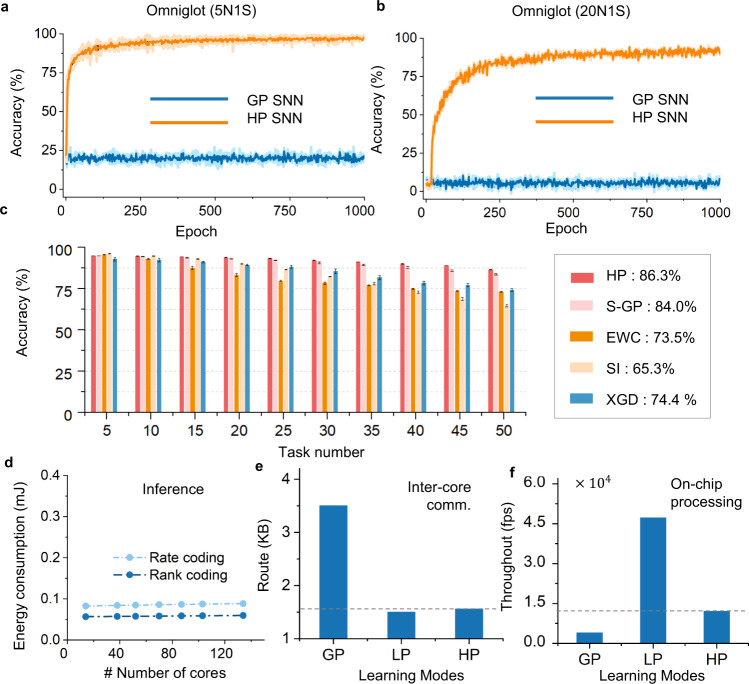
Performance evaluations of hybrid plasticity spiking neural networks. **a**, **b** Performance curves on the Omniglot dataset with five-way, one-shot (5N1S, **a**) and twenty-way, one-shot (20N1S, **b**) experiments. The average and standard deviation over five runs for each epoch are reported. **c** Histogram of the accuracy of different techniques using the shuffle-MNIST dataset. The right side shows the average performance of over 50 tasks. The sparsification-weight learning is called by the S-GP for short. The error bar is given by the standard deviation over five runs. Note that all models are based on spiking leaky integrate-and-fire neurons and slightly differ from that in other published work. **d** The HP model can support multiple spike coding schemes on the Tianjic and achieve highly efficient inference. Critically, the energy consumption increases slowly as the network size expands owing to the spike-based paradigm and local-memory structure. **e** The comparison of inter-core communication resources in three different learning modes. **f** The throughput evaluation of on-chip learning in three different learning modes. Details of hardware evaluation methods are described in the Methods.

Here, we used the Omniglot dataset to examine the performance of the proposed model. We adopted a widely used network structure^31,47,48^ to abstract features and compared their performance. We also fed the training labels to the last layer as used by ref. ^30^, during the presentation time so as to help the network establish an input-to-output correlation via the LP module. In this manner, when a query sample is received, the LP module may provide an augmented signal based on the correlation-based inter-product of the query sample and the centers of each previously appeared sample. Figure 5a, b depicts the comparison results. A detailed experimental setup is provided in Methods. Because vanilla backpropagation has difficulty in learning useful feature representations when given a limited number of samples, a single GP model is hardly learned in this task. The best accuracy of our model for five-way one-shot and twenty-way one-shot tasks is 98.7% and 94.6%, respectively, which are comparable with other state-of-art results and significantly higher than those of the previous SNNs. Compared with the single GP model, the improved accuracy indicates that the LP module plays a critical role in performance. In addition, without resorting to additional techniques, the synergic learning model can achieve competitive results that are comparable to the state-of-the-art results, as shown in Table 2.

**Table 2:** Comparison of the state-of-art results on Omniglot datasets.

Model	5-way 1-shot Acc. (%)	20-way 1-shot Acc. (%)
Human level^24^	–	95.5
MAML^47^	98.7	95.8
Non-spiking plastic nets^31^	98.3	–
Non-spiking Siamese nets^48^	97.3	88.2
Spiking LN^64^	83.8	–
Spiking nets with GP (this work)	28.4	8.5
Spiking nets with HP (this work)	98.7	94.6

### Continual learning

To further explore the benefits that the HP model might provide, we further investigate the ability of HP models for continual learning, that is, an ability to learn new tasks without forgetting the previous tasks^49^. Recent studies^50^ have shown that the motor cortex disinhibits a sparse subset of dendritic branches for new tasks, thereby reducing the disruption of network memory for previous tasks. It implies that the brain may multiplex some neuro-circuits while highly activating some synaptic connections to represent task-related information when solving new tasks. These motivate us to develop a distributed synergic learning paradigm, activating a sparse overlapping subset of weight connections by GP learning and modulating other synaptic connections by a task-sharing LP learning. Unlike the previous XdG method^25^ that uses a sub-network to solve a sub-task by masking parts of neurons in each task, our method uses a finer-grained synaptic modulation and a different synergic learning scheme. Here we allow the hybrid model to use a small number of overlapping connections to represent task-specific information, and LP learning to learn common features among tasks. By doing so, we expect to alleviate the disruption of network memory in different tasks and expand the learning capacity of the hybrid model to handle multiple tasks.

To this end, we examined the HP model performance on the standard shuffled MNIST dataset and compared it with a single GP model and the state-of-the-art results^25,51,52^. We ran all models five times and reported the testing results after fifty-task learning (Fig. 5c). We randomly activated 3% sparse and overlapping connections with the GP learning for each task and used the LP learning to learn other connections. The meta-parameters of local learning were trained using the first 35 tasks and fixed in the last 15 tasks for evaluation. A more detailed setting can be found in methods. Figure 5c indicates that during the 50-task learning, the HP model consistently achieves the best results compared with other works. The proposed model obviously outperforms the sparse GP model, which indicates the effectiveness of the proposed hybrid paradigm. In addition, because this paradigm allows the hybrid model to flexibly allocate different learning methods on different connections, the proposed model can leverage the many-core architecture to optimize the deployment of on-chip resources. We demonstrate the flexibility in the following section.

### Effectiveness analyses

We next analyze the effectiveness of the hybrid synergic learning model. Because the learning of such hybrid models is affected by both the external supervision error and internal synaptic behaviors, according to different learning circuits, we assume that the overall loss of the hybrid model can be decomposed into an explicit classification loss and an implicit loss driven by the network dynamics (see Methods). Then we remodel the local weight update from the perspective of the optimization, and analyze the effectiveness of the approximate regularization based on hetero-associative memory (HAM)^27,53^ and metric learning.

For the fault tolerance test, if we consider the local weight increment as a derivative of the implicit loss function, we can integrate the local weight increment and derive the implicit loss in a similar form as the energy function used in HAM^27,53^. Similarly, Hebbian-based operations can help to encode the previous patterns triggering neuron concurrent firing behaviors into a local minimum in the energy landscape. On the one hand, GP learning ensures that the network can selectively activate neuron firing and realize the correct response to input patterns. This indicates that the neuron concurrent firing behaviors are more likely to represent an adequate response for the previous training patterns. On the other hand, LP learning can gradually decrease the energy surface at every update based on the concurrent firing behaviors (Eq. (15)). Through implicitly optimizing this surface, local modules place an approximate regularization on the network structures. This encourages the network to selectively strengthen the weights triggering these concurrent firing activities and thereby produce a stronger stimulus for repeated or similar patterns. By combining LP and GP learning, the HP model can optimize the energy regularization and gradually relax the hierarchical representation of networks to local minimum states, which may encode the previous associative patterns, thereby exploiting the correlation embedded in the appeared training examples (see Methods).

We deliberate from the perspective of metric learning to discuss the model performance on few-shot learning. By clamping label signals into the local module, a correlation-based local module of the high-level input features and training labels can be established and a constraint can be placed with respect to the distribution of classes in the metric space. We prove that the local module can project an input pattern into a cosine-based embedding space and further produce a simple inductive bias by measuring the similarity between the query sample and the centers of each previously appeared sample (see Method). By doing so, the network is forced to learn from the embedding space representations to make the distance between samples within a class sufficiently small while the distance between samples from different classes is sufficiently large.

Through the above analysis, we demonstrate that the LP and GP learning complement each other to form the synergic learning model. An interesting finding is that we can adapt the hybrid model to different tasks only with minor modifications to the local modules. It provides another way for the design of loss functions. Considering that the brain prominently uses local learning to perform tasks, transferring a part of the design of loss functions to local modules is instructive and can bring benefits from at least two folds: (1) it can reduce the number of manually designed hyperparameters in the overall loss functions, such as converting the original regularized weighting coefficients to model the learning rates of the local module; (2) the LP-based operation endows a network with online and high-parallelism properties, which especially facilitates the implementation on many-core neuromorphic hardware by the fine-grained parallelism architecture (see Discussion).

### Hybrid computation on the Tianjic by algorithm-hardware co-design

By implementing our model on the Tianjic, next, we demonstrate that the proposed hybrid model is well suitable for implementing in the dedicated neuromorphic hardware. We first instantiated our model on the Tianjic neuromorphic chips (see Methods) and evaluated the efficiency of on-chip inferences. As indicated in Fig. 5d, the proposed HP model can flexibly support the rate-based and temporal-based spiking coding schemes and meet different on-chip inference requirements for accuracy and inference latency. As the network size increases, owing to the spike-based paradigm and local-memory structure, the energy consumption scales very slowly. More importantly, by virtue of the neuromorphic many-core architecture, the model can alleviate the overall power consumption and achieve speeds that are orders of magnitude faster than the general-purpose computer (Supplementary Table 1).

Due to the different weight update manner, the LP and GP learning complement each other in the computational resources. This property can be leveraged by the massive parallelism of many-core hardware for on-chip learning. Since by far there is no reported solution that can simultaneously support LP and GP approaches on many-core chips, we exploited the method of algorithm-hardware co-designs and designed a hybrid on-chip learning scheme through developing an online learning mapping scheme with a new cycle-accurate hardware simulator and a mapping scheme (see Methods), thereby evaluating the computational resources of on-chip hybrid learning. We evaluated the hardware efficiency using continual multitask learning. During this process, we mainly adopted four steps for the entire evaluation, including mapping design scheme, software tool configuration, simulating on-chip running process, and data arrangement (see Methods). Figure 5e, f exhibits the simulation results of the routes and throughput of implementing the LP, GP, and HP models on Tianjic chips. The proposed hybrid approach allows the flexible configuration of GP and LP learning on different connections. Since only a small number of weight connections are used to receive task-specific supervision signals, the workloads of inter-core communications on many-core architectures can be significantly alleviated (Fig. 5e), and the local learning can be further deployed in core-in resources by utilizing the decentralized many-core architecture^13,54^. With the highly parallel and near-memory computing architecture of neuromorphic architecture, it can efficiently realize local learning and facilitate on-chip learning with high throughput (Fig. 5f).

In addition, as indicated in Fig.5e, f, the forward and backward dataflow of the GP circuit cannot make full use of the pipelined processing mechanism of many-core chips. Thus, how to efficiently optimize the implementation of the GP circuit in the neuromorphic chips is an important direction to improve the overall efficiency further. Alternatively, some emerging neuromorphic hardware, such as the Loihi^13^, have embedded part of X86 cores in one single chip, which may provide a potential candidate for implementing GP in chips in the future. Thus, the combination of algorithm-hardware co-design is also a feasible direction to further develop a hybrid computing paradigm. For example, the main body of many-core structure can be used to perform local learning, while the embedded microprocessors are used to perform GP learning, which may improve hybrid on-chip efficiency and promote applications of the hybrid model.

## Discussion

In this work, we reported a spike-based hybrid model that endows SNNs with an efficient synergic learning capability for handling multiple learning scenarios. Guided by the hippocampal plasticity mechanism, we developed a brain-inspired meta-learning paradigm to integrate these two types of learning and further explore multiple synergic learning strategies that can quickly adapt the synergic learning model for solving different learning scenarios. Our results indicate that with small modifications of local modules, the hybrid synergic learning model can achieve significantly higher performances than single-learning models on sequential classification tasks, and three different learning scenarios. Finally, we implemented the model in the Tianjic neuromorphic platform by exploiting algorithm-hardware co-designs and demonstrated the advantages of the proposed hybrid model on neuromorphic chips.

Understanding how the interconnected neurons in the brain combine top-down modulation information and bottom-up local information to learn to solve tasks is an active research area in both neuroscience and machine learning. The related surrogate gradient methods^8,42^ use the continuous relaxation of the gradients and provide a differentiable spiking network to update weights in a fully local computation. The e-prop^7^ algorithm combines the top-down supervision signals and local eligibility traces to approximate the backpropagation of signals through time. Unlike the previous studies, we use a meta-learning method to design the synergic learning circuit. We model the hyperparameters of local learning as a special type of meta-parameters that can be modified by top-down supervision signals and indirectly influence the behaviors of synapse plasticity. Furthermore, by deriving the implicit loss function from the LP, we prove that the local modules can act as a regularization over the network topology and temporal dynamics, indicating that the roles of LP and GP circuits are different from the perspective of optimization. We deliberate from the associative memory and metric learning as an illustration and demonstrate that by constructing different correlation-based modules, the hybrid synergic learning model can be linked with several existing powerful learning algorithms, thereby providing support for the effectiveness analysis.

Some meta-learning-based global-local learning methods have been developed for non-spiking models. In the early 1990s, ref. ^29^ has proposed a basic framework that can optimize the parameterized local learning rule by global supervision signals. Several recent work^31,55^ use trainable connection weights of local modules and established large-scale non-SNNs. We adopt the basic idea of learnable LP as in refs. ^29,31,55^. One main difference from the previous works is that we optimize underlying weights and LP by the bilevel optimization, and develop a configurable hybrid strategy that can exploit synergic learning in different ways with greater flexibilities in solving many other learning scenarios. More importantly, we incorporate various spiking dynamics for establishing hybrid neuromorphic models and exploit the algorithm-and-hardware co-designs to develop feasible hardware implementations for online hybrid learning. Through the combination with neuromorphic computing, many unreported advantages of global-local learning are revealed, such as the fault-tolerance on neuromorphic sensors and the complementary computational efficiency on neuromorphic chips.

A salient feature of this work is the formulation of spike-based neuromorphic models. In principle, SNN is a special type of neural network that memorizes historical temporal information via intrinsic neuronal dynamics and encodes information into spike trains, thereby enabling event-driven energy-efficiency computation. They are suitable for scenarios with rich spatio-temporal event-driven information and sparse dataflow and have powered many applications in neuromorphic sensors and neuromorphic chips^28,56^. By deriving from neuronal dynamics and incorporating various dynamic behaviors of spiking neurons, our model retains many prominent biological attributes and provides a general method to meta-learn spike-based LP (Supplementary Note 4). By introducing complementary learning features, the proposed synergic learning show promises in improving information processing on NVSs. Besides that, since the neuromorphic chips can leverage the asynchronous spike-based communication and high-parallel computation of local learning, the proposed hybrid model provides an opportunity to promote efficient hardware implementation and facilitate the exploration of the hybrid computing paradigm in the neuromorphic hardware platform. ref. ^57^ reports an online functional-level simulation scheme of meta-learning models on Loihi chips. However, because the functional-level simulation simplifies the model performance environment and loses many underlying fine-grained execution details related to the hardware environment, it is difficult to accurately evaluate the actual consumption and practical advantages of hybrid learning on chips. Conversely, we implement the hybrid model in the Tianjic by developing the new cycle-accurate hardware simulator and mapping scheme. It can prompt algorithm-hardware co-designs and the exploration of hybrid computing paradigms on neuromorphic architecture.

In summary, the neuromorphic synergic learning model developed in this work exhibits a superior learning ability for solving multiple different learning tasks and the excellent energy efficiency of the hybrid computation paradigm on neuromorphic chips, which may open an avenue for the collaborative development of neuromorphic algorithms and neuromorphic computing chips.

## Methods

### Model establishment

The hybrid plastic approach is based on two sets of differential equations. The first set describes the membrane potential dynamics as follows:

2 $${\tau }_{u}\frac{d{u}_{i}}{dt}=-{u}_{i}(t)+{\sum }_{j=1}^{{l}_{n}}{w}_{ij}(t){s}_{j}(t),$$

3 $${s}_{j}(t)={\sum }_{{t}_{j}^{f} < t}\delta (t-{t}_{j}^{f}),$$

where ${w}_{ij}$ denotes the weight of the synapse connecting pre-neuron *j* and post-neuron *i*, ${u}_{i}$ denotes the membrane potential of neuron *i*, ${\tau }_{u}$ denotes the membrane time constant, ${s}_{j}({{{{{\rm{t}}}}}})$ denotes the afferent spike trains, ${t}_{j}^{f}$ denotes the firing time, and ${l}_{n}$ denotes the number of neurons in the ${l}_{th}$ layer.

The second set establishes on a type of diffusion dynamics of ion channels^58^, modeled by

4 $${\tau }_{w}\frac{d{w}_{ij}}{dt}={w}_{ij}^{g}-{w}_{ij}(t)+P(t,\,pr{e}_{j}(t),pos{t}_{i}(t);\theta ),$$

where ${\tau }_{w}$ denotes the synaptic constant. The first term ${w}_{ij}^{g}-{w}_{ij}(t)\,$ in the right of Eq. (4) denotes the recovery of ${w}_{ij}(t)$ into a ground state ${w}_{ij}^{g}$, in which we set zero in the experiments. The second term $P(\ast )$ represents general LP controlled by presynaptic spike activity, $pr{e}_{j}(t)\;\triangleq\; \{{s}_{j}(t)\}$, postsynaptic spike activity, $pos{t}_{i}(t)\;\triangleq\; \{{u}_{i}(t),{s}_{i}(t)\}$, and a group of layer-sharing controllable factors ${{{{{\boldsymbol{\theta }}}}}}$ that includes the local learning rate, sliding threshold, and other hyperparameters that are determined by the specific local learning rules (see below).

As $P(\ast )$ is generic for modeling local learning rule, here we take a specific expression, a variant of Hebbian rule which is formulized by

5 $$P\;\triangleq\; {k}^{corr}{s}_{j}(t)(\rho ({u}_{i}(t))+{\beta }_{i}),$$

where ${k}^{corr}$ is a weight hyperparameter, $\rho (x)$ is a bounded nonlinear function, and ${\beta }_{i}\le 0$ is an optional sliding threshold to control weight change directions and prevent weight explosions. It, therefore, can update the weight according to concurrent presynaptic firing and postsynaptic membrane activity. Integrating Eq. (4), we get

6 $${w}_{ij}(t)={w}_{ij}({t}_{n0}){e}^{-\frac{t-{t}_{n0}}{{\tau }_{w}}}+{\int }_{{t}_{n0}}^{t}P(x,pr{e}_{j}(x),pos{t}_{i}(x);{{{{{\boldsymbol{\theta }}}}}}){e}^{-\frac{t-x}{{\tau }_{w}}}dx,$$

where ${w}_{ij}({t}_{n0})$ denotes the instantaneous phasic state of synaptic weight at the phase time ${t}_{n0}$. Because the HP approach uses a potential trajectory rather than an equilibrium state for computation, the dependence on the initial parameter ${w}_{ij}({t}_{n0})$ is non-trivial. Based on it, we assume that the HP approach can perform supervised learning through modifying the phasic weight values ${w}_{ij}({t}_{n0})$ at a certain time ${t}_{n0}$, in a form of the instantaneous top-down modulated signal. Consequently, we substitute the synaptic Eq. (6) into the membrane potential Eq. (2) by

7 $${{{{{{\rm{\tau }}}}}}}_{u}\frac{d{u}_{i}}{dt}=-{u}_{i}(t)+\mathop{\sum }\limits_{j=1}^{{l}_{n}}{s}_{j}(t){w}_{ij}({t}_{n0}){e}^{-\frac{t-{t}_{n0}}{{\tau }_{w}}}+\mathop{\sum }\limits_{j=1}^{{l}_{n}}({\int }_{{t}_{n0}}^{t}P(x,pr{e}_{j}(x),pos{t}_{i}(x);{{{{{\boldsymbol{\theta }}}}}}){e}^{-\frac{t-x}{{\tau }_{w}}}){s}_{j}(t)dx.$$

To enable the continuous dynamics compatible with backpropagation, we use a modified Euler method to get an explicit iterative version of Eq. (7)

8 $$\begin{array}{c}{{{{{{\rm{\tau }}}}}}}_{{{{{{\rm{u}}}}}}}\frac{{u}_{i}({t}_{m+1})-{u}_{i}({t}_{m})}{dt}=-{u}_{i}({t}_{m})+{\sum }_{j=1}^{{l}_{n}}{s}_{j}({t}_{m}){w}_{ij}({t}_{n0}){e}^{-\frac{{t}_{m}-{t}_{n0}}{{\tau }_{w}}}\\ \,+{\sum }_{j=1}^{{l}_{n}}{s}_{j}({t}_{m}){\int }_{{t}_{n0}}^{{t}_{m}}P(x,pr{e}_{j}(x),pos{t}_{i}(x);{{{{{\boldsymbol{\theta }}}}}})\,{e}^{-\frac{{t}_{m}-x}{{\tau }_{w}}}dx,\end{array}$$

where we use ${t}_{m}$ to refer to the simulation timestep. Sorting the formula and substituting the specific expression of $P(x)$, we get a set of final signal propagation equations as follows

9 $$\left\{\begin{array}{c}{u}_{i}^{l}({t}_{m+1})=(1-{s}_{i}^{l}({t}_{m}))(1-{k}_{u}){u}_{i}^{l}({t}_{m})+{k}_{u}{\sum }_{j=1}^{{l}_{n}}{s}_{j}^{l-1}({t}_{m}){w}_{ij}^{l}({t}_{n0}){e}^{-\frac{{t}_{m}-{t}_{n0}}{{\tau }_{w}}}+\\ \,{k}_{u}{\sum }_{j=1}^{{l}_{n}}{s}_{j}^{l-1}({t}_{m}){\sum }_{{t}_{i}^{f},{t}_{j}^{f} < {t}_{m}}{k}_{ij}^{l,corr}{H}_{j}^{l-1}({t}_{m}-{t}_{j}^{f})(\rho ({u}_{i}({t}_{m})))+{\beta }_{i}\big){e}^{-\frac{{t}_{m}-{t}_{j}^{f}}{{\tau }_{w}}},\\ {s}_{i}^{l}({t}_{m})=H({u}_{i}^{l}({t}_{m})-{v}_{th}),\end{array}\right.$$

where ${k}_{u}\;\triangleq\; \frac{dt}{{\tau }_{u}}$, the upper index *l* denotes the ${l}_{th}$ layer, and $H(x)$ is the firing function determined by the Heaviside function. Specifically, if ${u}_{i}^{l}({t}_{m})$ exceeds the threshold ${v}_{th}$, $H(x)=1$; otherwise $H(x)=0.$ Regarding the non-differentiable points of spike firing function $H(x)$, we use the surrogate gradient methods proposed by refs. ^8,42^ and adopt a suitable rectangle function^59^ for approximating the derivative of the spiking function. In addition, we multiply the gated signal $(1-{s}_{i}^{l})$ in the membrane attenuation term $(1-{k}_{u}){u}_{i}^{l}({t}_{m})$ to realize the firing-and-resetting behavior of spiking dynamics.

Finally, to make the expression clearly, we replace the summation of local activity into an iterative variable ${P}_{ij}^{l}({t}_{m})$, relax ${k}_{ij}^{l,corr}\,$by two elastic regular factors, ${k}_{ij}^{l,corr}={\alpha }_{i}^{l}{\eta }_{j}^{l}$, and transform Eq. (8) into an iterative version,

10 $$\left\{\begin{array}{c}{u}_{i}^{l}({t}_{m})=(1-{s}_{i}^{l}({t}_{m-1}))(1-{k}_{u}){u}_{i}^{l}({t}_{m-1})+{k}_{u}\mathop{\sum }\limits_{j=1}^{{l}_{n}}\,\left({w}_{ij}^{l}({t}_{n0}){e}^{\frac{{t}_{n0}-{t}_{m}}{{\tau }_{w}}}+{\alpha }_{i}^{l}{P}_{ij}^{l}({t}_{m})\right){s}_{j}^{l-1}({t}_{m}),\\ \,{P}_{ij}^{l}({t}_{m})={P}_{ij}^{l}({t}_{m-1}){e}^{-\frac{dt}{{\tau }_{w}}}+{\eta }_{j}^{l}{s}_{j}^{l-1}({t}_{m})(\rho ({u}_{i}^{l})+{\beta }_{i}^{l}),\\ \,{s}_{i}^{l}({t}_{m})\,=H({u}_{i}^{l}({t}_{m})-{v}_{th}),\end{array}\right.,$$

where $dt$ denotes the length of timestep,$\,{\alpha }^{l}\,$controls the impact of local modules and ${\eta }_{j}^{l}$ controls the local learning rate. It therefore formulizes the meta modules ${{{{{{\boldsymbol{\theta }}}}}}}^{l}$ as a group of layer-wise parameters {${\alpha }^{l},{\eta }^{l},\,{\beta }^{l}$}.

For classification output, we take the one-hot encoding and use $N$ neurons of the output layer to represent classification results. Then we incorporate different spike coding schemes into a general framework and describe the classification loss function $C$ by

11 $$C({{{{{\bf{w}}}}}},{{{{{\boldsymbol{\theta }}}}}})\;\triangleq\; C\left({{{{{\bf{y}}}}}},\mathop{\sum }\limits_{m=1}^{T}{z}_{{t}_{m}}q({{{{{{\bf{u}}}}}}}^{{n}_{l}}({t}_{m}))\right),$$

where ${{{{{\bf{y}}}}}}$ is the ground truth, ${n}_{l}\,$ is the number of layers, $\,T$ denotes the simulation windows, $C$ is a classification loss, $q(x)$ is a non-decreasing bounded function depending on specific coding schemes, ${z}_{{t}_{m}}\in {R}_{\ge 0}$ is the weight associated with timestep ${t}_{m}$. This formulization can adapt to the rate-based coding when ${z}_{{t}_{m}}=1/T$, $q(x)=H(x-{v}_{th})$, and adapt to rank-based coding when ${z}_{{t}_{m}}=1[{t}_{m}=T].$

Given the signal propagation Eq. (10) and a specific decoding scheme, ideally, we can search the optimal value of network parameters, ${{{{{\boldsymbol{\theta }}}}}}$ and ${{{{{\bf{w}}}}}}$, using the BPTT algorithm. Because the parameter ${{{{{\boldsymbol{\theta }}}}}}$ is a higher-level variable to modulate weights, we exclusively establish the optimization of ${{{{{\boldsymbol{\theta }}}}}}$ and formulize a general expression as follows:

12 $${\min \,\tilde{C}}_{{{{{{\boldsymbol{\theta }}}}}}}\;\triangleq\; \mathop{\sum}\limits_{{\pi }_{i}\in \varGamma }{C}_{{\pi }_{i}}^{val}\,({{{{{{\bf{w}}}}}}}{\ast }({{{{{\boldsymbol{\theta }}}}}}),{{{{{\boldsymbol{\theta }}}}}}),\qquad{{{{{\rm{s}}}}}}.{{{{{\rm{t}}}}}}.,{{{{{{\bf{w}}}}}}}{\ast }(\theta )={{{\mbox{arg}}}}\mathop{{{\min }}}\limits_{{{{{{\bf{w}}}}}}}{C}_{{\pi }_{i}}^{train}({{{{{{\bf{w}}}}}}}{\ast },{{{{{\boldsymbol{\theta }}}}}}).$$

The task ${\pi }_{i}\triangleq \{C({{{{{\bf{y}}}}}},{{{{{\bf{x}}}}}},{{{{{\bf{w}}}}}}|{{{{{\boldsymbol{\theta }}}}}})\}$ samples from the task distribution set $\varGamma ,$ and consists of a certain loss function *C* and a set of training data and validation data. Here we use ${C}_{{\pi }_{i}}^{val}$ and ${C}_{{\pi }_{i}}^{train}$ to distinguish the loss function of training data and validation data in few-shot learning and multitask learning. In Eq. (12), obtaining precision solutions of ${{{{{{\bf{w}}}}}}}^{\ast }$ is usually prohibitive and computationally expensive. To ensure convergence and obtain feasible solutions, we formulize the above problem as a bilevel optimization that is usually used for optimizing two associated upper-level and lower-level variables^41,47,60^. To this end, we follow the work^47,60^ to approximate the ${{{{{{\bf{w}}}}}}}^{\ast }$ by one-step gradient update in one training batch. After updating the weight ${{{{{\bf{w}}}}}}$, we alternatively update$\,{{{{{\boldsymbol{\theta }}}}}}$ across a validation task batch using the updated weights to learn fast adaptation of LP. In this manner, the optimization can be divided into two parts by iteratively optimizing parameters ${{{{{\bf{w}}}}}}$ and ${{{{{\boldsymbol{\theta }}}}}}$. More concretely, in the *k* iteration, we sample a training task batch and updated the weights ${{{{{{\bf{w}}}}}}}_{k}$ by the BPTT. Then we sample a validation task batch using gradient updates ${{{{{{\bf{w}}}}}}}_{k}\,$ and optimize the ${{{{{{\boldsymbol{\theta }}}}}}}_{k}$ over task batch by the following Eq. (13) until the training converges

13 $${\nabla }_{{{{{{{\boldsymbol{\theta }}}}}}}_{k}}\tilde{C}=\mathop{\sum}\limits_{{\pi }_{i}\in {\varGamma }_{s}\,}{\nabla }_{{{{{{{\boldsymbol{\theta }}}}}}}_{k}}{C}_{{\pi }_{i}}^{val}({{{{{{\bf{w}}}}}}}^{\ast },\,{{{{{{\boldsymbol{\theta }}}}}}}_{k})\approx \mathop{\sum}\limits_{{\pi }_{i}\in {\varGamma }_{s}\,}{\nabla }_{{{{{{\boldsymbol{\theta }}}}}}}{C}_{{\pi }_{i}}^{val}({{{{{{\bf{w}}}}}}}_{k}-\xi {\nabla }_{{{{{{\bf{w}}}}}}}{C}_{{\pi }_{i}}^{train}({{{{{{\bf{w}}}}}}}_{k-1},{{{{{{\boldsymbol{\theta }}}}}}}_{k-1}),\,{{{{{{\boldsymbol{\theta }}}}}}}_{k}),$$

where ${\varGamma }_{s}$ is a task batch set sampled from the task distribution set $\varGamma$, $\xi \ge 0$ is the learning rate of approximation weight updates that can be used for accelerating convergence^60^.

### Support rank ordering coding

By deriving from a type of ion-channel dynamics model, our model maintains the synaptic decay dynamics, $k(t)={e}^{\frac{{t}_{n}-t}{{\tau }_{w}}}$, during information transmission. Because the presynaptic spike signals must be filtered by the temporal filtering $k(t)$ to the postsynaptic neurons, it implies that the arriving time affects the information transition. On this basis, we find a potential relationship between the HP model and the classic rank order coding assumption (see Supplementary Note 3), and develop an evidence-accumulation temporal decoding scheme. Formally, as long as the first spike is triggered by the winning neuron in the output layer, the HP SNNs will stop signal inference and produce results based on the index of the winning neuron. Then, the scaled membrane potential of the output neurons is used as an output representation to calculate the loss. In this manner, in addition to supporting a conventional rate-base decoding scheme, we can utilize the synaptic dynamics and threshold mechanism of spiking neurons to implement an event-driven inference mode.

### Implicit loss function

We consider the impact of local modules as a form of implicit loss and analyze the effectiveness from the perspective of optimization. Because the learning process of the HP model is affected by the supervision signals and internal dynamics, accordingly, its overall loss function $E$ is more likely not only incorporating an explicit classification loss $C$, but also building on an implicit loss function ${E}_{in}$ generated by the inherent dynamics of the network. According to the different learning circuits, we first make the decomposability assumption on the general overall loss as below:

14 $$E\;\triangleq\; \,C(t,{{{{{\bf{x}}}}}},{{{{{\bf{y}}}}}};{\lambda }_{1}{{{{{{\bf{w}}}}}}}_{GP,t},{\lambda }_{2}{{{{{{\bf{w}}}}}}}_{LP,t},{{{{{\boldsymbol{\theta }}}}}})+{\lambda }_{3}{E}_{in}(t,{\{pr{e}^{l},pos{t}^{l},{{{{{{\bf{w}}}}}}}^{l},{{{{{{\boldsymbol{\theta }}}}}}}^{l}\}}_{l=2}^{{n}_{l}}),$$

where ${{{{{\bf{x}}}}}}\,$ and ${{{{{\bf{y}}}}}}$ are external input data, ${n}_{l}$ denotes the total number of layers, and ${{{{{{\rm{\lambda }}}}}}}_{1,2,3}\in {R}_{\ge 0}$ denote the influential factor of each part. We follow the notations of Eq. (1) and in a slight abuse of notation, we explicitly express the composition ${\lambda }_{1}{{{{{{\bf{w}}}}}}}_{GP,t},{\lambda }_{2}{{{{{{\bf{w}}}}}}}_{LP,t}$ on $C$ to highlight the difference between the hybrid model and single-learning-based model. The expression of $E$ can be regarded as an extension of a single-learning-based network. In the case of ${\lambda }_{2}={\lambda }_{3}=0$, $E$ degenerates to the conventional classification loss for the GP-based network, and in the case of ${\lambda }_{1}=0$, the network reduces to the LP-based network. We use the implicit function to analyze the effectiveness of HP models on fault-tolerance learning and few-shot learning.

### Effectiveness analyses on fault-tolerance learning

If we treat the local weight increment as an implicit derivative for a part of the overall loss function *E*, it inspires us to integrate its weight increment to obtain the implicit loss function ${E}_{in}$. For simplicity, we mainly focus on the impact of local weight rules on the optimization of the current layer weight ***w*** and illustrate the effectiveness of local modules for the specific task.

In the fault tolerance learning, we accordingly treat the local weight increment $\varDelta {w}_{ij,t}^{l}$ as the implicit derivative of ${E}_{in}^{l}$ by

15 $$\frac{\partial {E}_{in}}{\partial {w}_{ij}^{l}}\propto -\varDelta {w}_{ij,t}^{l}\propto -{s}_{j,t}^{l-1}\rho ({u}_{i,t}^{l}).$$

We use the Hebbian rule and set $\rho ({u}_{i,t}^{l})=H({u}_{i,t}^{l}-{v}_{th})$ in the derivation. Integrating the above equation, we can get a loss expression ${E}_{in}$ as follows:

16 $${E}_{in}\approx -{\sum }_{t=1}^{T}{\sum }_{l=2}^{{n}_{l}}{{{{{{\bf{s}}}}}}}_{t}^{l-1T}\int H({{{{{{\bf{u}}}}}}}_{t}^{l}-{v}_{th})d{{{{{{\bf{w}}}}}}}^{l}=-{\sum }_{t=1}^{T}{\sum }_{l=2}^{{n}_{l}}{{{{{{\bf{s}}}}}}}_{t}^{l-1^{T}}({{{{{{\bf{w}}}}}}}_{t}^{l}{{{{{{\bf{s}}}}}}}_{t}^{l}-\int {{{{{{\bf{w}}}}}}}^{l}\,\frac{dH}{d{{{{{{\bf{w}}}}}}}^{l}}),$$

Since the derivative of Heaviside function is zero for $u\;\ne\; {v}_{th}$, the following equation holds

17 $${E}_{in}\approx -{\sum }_{t=1}^{T}{\sum }_{l=2}^{{n}_{l}}{{{{{{\boldsymbol{s}}}}}}}_{t}^{l-1^{T}}{{{{{{\boldsymbol{w}}}}}}}_{t}^{l}{{{{{{\boldsymbol{s}}}}}}}_{t}^{l},(u\;\ne\; {v}_{th}).$$

We note that a form of the loss function in the Eq. (17) is similar to the energy function used in HAM^27,53,61^ in which Hebbian-based operations help networks encode the previous associative patterns into a local minimum of the energy surface. It inspires us to explain the model effectiveness from the optimization of energy function. Specifically, in the HP models, the GP-based learning ensures that the network can selectively activate parts of neurons firing and realizes a correct response to input patterns. Thus, the associative patterns of neuron concurrent firing behaviors (i.e., ${{{{{{\bf{s}}}}}}}_{t}^{l-1^{T}}{{{{{{\bf{s}}}}}}}_{t}^{l}$) are more likely to represent an effective response for input patterns. On the other hand, as shown in Eq. (17), Hebbian-based operation can decrease the surface at every update. Therefore, by optimizing the energy surface, the local module places an approximate regularization on the network structures with the punishment of $-{\sum }_{l=2}^{{n}_{l}}{{{{{{\bf{s}}}}}}}_{t}^{l-1^{T}}{{{{{{\bf{w}}}}}}}_{t}^{l}{{{{{{\bf{s}}}}}}}_{t}^{l}$. It encourages the network to strengthen the weights triggering neuron concurrent firing behaviors, resulting in a stronger stimulus for similar or repeated patterns. Collectively, by combing the LP and GP methods, the HP model can relax the hierarchical representation of the networks to local minimum states that are more likely to encode an effective response to the previous associative patterns, thereby exploiting the correlation embedded in the appeared training patterns for the recognition of incomplete patterns.

Please note that unlike HAM models using one or more bi-directional iterations for pattern reconstruction^27,53^, the HP model leverages the memory matrix for the classification of disturbance patterns. It can be further illustrated by analyzing the correlation-based augmented information of local modules. Given a general input and output dataset $D={\{({{{{{{\bf{x}}}}}}}_{i},{{{{{{\bf{y}}}}}}}_{i})\}}_{i}^{N}$, where ${{{{{{\bf{y}}}}}}}_{i}\in {R}^{m\times 1}$ refers to the response of the current layer to the input ${{{{{{\bf{x}}}}}}}_{i}$, let us assume that a querying sample $\tilde{{{{{{\bf{x}}}}}}}$ $\in {R}^{n\times 1}$ is received and belongs to the ${D}_{k}$ category. Then the local module produces an augmented information ${{{{{{\bf{I}}}}}}}_{LP}$ by

18 $${{{{{{\boldsymbol{I}}}}}}}_{LP}={{{{{{\boldsymbol{w}}}}}}}_{LP}\tilde{{{{{{\boldsymbol{x}}}}}}}={\sum }_{j}{{{{{{\boldsymbol{y}}}}}}}_{j}{{{{{{\boldsymbol{x}}}}}}}_{j}^{T}\tilde{{{{{{\boldsymbol{x}}}}}}}={\sum }_{{{{{{{\boldsymbol{x}}}}}}}_{k}\in {D}_{k}}{{{{{{\boldsymbol{y}}}}}}}_{k}({{{{{{\boldsymbol{x}}}}}}}_{k}^{T}\tilde{{{{{{\boldsymbol{x}}}}}}})+{\sum }_{{x}_{j}\notin {D}_{k}}{{{{{{\boldsymbol{y}}}}}}}_{j}({{{{{{\boldsymbol{x}}}}}}}_{j}^{T}\tilde{{{{{{\boldsymbol{x}}}}}}}).$$

When the network receives a disturbance sample $\tilde{{{{{{\bf{x}}}}}}}$, ${w}_{LP}$ provides the augmented information of the previously stored pattern ${{{{{{\bf{y}}}}}}}_{i}$ in the form of a weighting coefficient $({{{{{{\bf{x}}}}}}}_{k}^{T}\tilde{{{{{{\bf{x}}}}}}})$. Since the inner product from the same class can provide stronger augmented information, it also indicates that the local module can exploit the correlation between the input sample and the previous appeared associative patterns, thereby facilitating the classification of disturbance patterns.

### Effectiveness analyses on few-shot learning

Next, we show that the correlation-based local module can place a constraint with respect to the distribution of classes in the cosine-based metric space to accelerate convergence. Assume that we have a set of training samples $D\,=\,{\{({{{{{{\bf{x}}}}}}}_{k},{{{{{{\bf{y}}}}}}}_{k})\}}_{k=1}^{{N}_{D}}$ where ${{{{{{\bf{x}}}}}}}_{{{{{{\rm{k}}}}}}}\in {R}^{m\times 1}$ is an *m*-dimensional feature vector, ${{{{{{\bf{y}}}}}}}_{k}\in {R}^{n\times 1}$ is the one-hot label, ${N}_{D}$ denotes the sample number of dataset *D*. We refer ${{{{{{\bf{x}}}}}}}_{k}$ to the general features coming from raw data or the last ${n}_{l}-1$ layer. By introducing the training labels to the output neurons, the local module constructs a Hebbian-like matrix as follows:

19 $${{{{{{\bf{w}}}}}}}_{LP}={\sum }_{k\in {N}_{D}}{\eta }_{k}{{{{{{\bf{y}}}}}}}_{k}{{{{{{\bf{x}}}}}}}_{k}^{T}={\sum }_{k=1}^{K}({{{{{{\bf{y}}}}}}}_{k}{\sum }_{i=1}^{{N}_{{D}_{k}}}{\eta }_{i}{{{{{{\bf{x}}}}}}}_{i}^{T})={\sum }_{k=1}^{K}{{{{{{\bf{y}}}}}}}_{k}{{{{{{\bf{c}}}}}}}_{k}^{T},$$

where *K* denotes the class number of $D$ and ${D}_{k}$ denotes the subset of examples within the same class. Here we set the learning rate ${\eta }_{i}=\frac{1}{{N}_{{D}_{k}}\left\|{{\bf{x}}}_{i}\right\|_2}$ and refer ${{{{{{\bf{c}}}}}}}_{k}=\frac{1}{{N}_{{D}_{k}}}{\sum }_{i=1}^{{N}_{{D}_{k}}}\frac{{{{{{{\bf{x}}}}}}}_{i}}{\left\|{{\bf{x}}}_{i}\right\|_2}$ to the sample mean of the class ${D}_{k}$. To keep the clarity of proof, we also simplify the modeling of other meta-parameters. Based on Eq. (18), when entering a query sample $\tilde{{{{{{\bf{x}}}}}}}$, the local module produces an inductive bias ${{{{{{\bf{I}}}}}}}_{LP}({{{{{\boldsymbol{x}}}}}})\,$ with intensity ${\sum }_{k=1}^{K}{{{{{{\rm{y}}}}}}}_{k}({{{{{{\boldsymbol{c}}}}}}}_{k}^{T}\tilde{{{{{{\bf{x}}}}}}}).\,$ Then the membrane potential of output neuron is governed by

20 $${u}_{i}^{{n}_{l}}({t}_{m})={\lambda }_{1}{I}_{GP,i}({t}_{m})+(1-{\lambda }_{1}){I}_{LP,i}({t}_{m})={\lambda }_{1}{\sum }_{j=1}^{{l}_{n}-1}{w}_{ij}\tilde{{x}_{j}}({t}_{m})+(1-{\lambda }_{1}){\sum }_{k=1}^{K}{y}_{k,i}{{{{{{\bf{c}}}}}}}_{k}\tilde{{{{{{\bf{x}}}}}}}({t}_{m}),$$

where ${y}_{k,i}$ is the $i$ element of the one-hot label ${{{{{{\bf{y}}}}}}}_{k}$, which satisfies that ${y}_{k,i}={1}_{\{k\}}(i)$. In the experiment, we initialize ${{{{{{\rm{\lambda }}}}}}}_{1}\,$with a small positive value to strengthen the impact of ${{{{{{\bf{I}}}}}}}_{LP}$ in the early training phase. In this manner, we can give an intuitive interpretation for the inductive bias ${{{{{{\bf{I}}}}}}}_{LP}$ from the Euclidean distance $L$ between the label ${{{{{\bf{y}}}}}}$ and ${{{{{{\bf{u}}}}}}}^{{l}_{n}}$. Assuming that the input pattern belongs to $i\,$ class, $L$ can be calculated by

21 $$\begin{array}{c}L({{{{{{\bf{u}}}}}}}^{{l}_{n}},{{{{{\bf{y}}}}}})=\mathop{\sum}\limits_{q\ne i}{({\lambda }_{1}{I}_{GP,q}+(1-{\lambda }_{1})({{{{{{\bf{c}}}}}}}_{q}^{T}\tilde{{{{{{\bf{x}}}}}}}))}^{2}+{\left(1-(({\lambda }_{1}{I}_{GP,i}+(1-{\lambda }_{1})({{{{{{\bf{c}}}}}}}_{i}^{T}\tilde{{{{{{\bf{x}}}}}}})))\right)}^{2}\\ \qquad={\sum }_{q\ne i}{({\lambda }_{1}{I}_{GP,q}+(1-{\lambda }_{1}){{{{{{\bf{c}}}}}}}_{q}^{T}\tilde{{{{{{\bf{x}}}}}}})}^{2}+{(1-{{{{{{\bf{c}}}}}}}_{i}^{T}\tilde{{{{{{\bf{x}}}}}}}-{\lambda }_{1}(({I}_{GP,i}-{{{{{{\bf{c}}}}}}}_{i}^{T}\tilde{{{{{{\bf{x}}}}}}})))}^{2},\end{array}$$

where we set $T\,=1$ and omit the index *t* for neatness. Note that the ${{{{{{\rm{\lambda }}}}}}}_{1}$ is a pre-defined small amount, thus, the ${L}_{\phi }$ has the two main components ${(1-{c}_{i}^{T}\tilde{{{{{{\bf{x}}}}}}})}^{2}$and ${(1-{{{{{{\rm{\lambda }}}}}}}_{1})}^{2}{{{{{{\bf{c}}}}}}}_{q}^{T}\tilde{{{{{{\bf{x}}}}}}}$. To minimize the distance $L$, the network is forced to learn the specific feature mapping that projects the distance between samples within a class sufficiently small (by the punishment of ${(1-{{{{{{\bf{c}}}}}}}_{i}^{T}\tilde{{{{{{\bf{x}}}}}}})}^{2}$) and the distance between samples from different classes sufficiently large (by the punishment of ${{{{{{\bf{c}}}}}}}_{q}^{T}\tilde{{{{{{\bf{x}}}}}}}$)). In this manner, the inductive bias ${{{{{{\bf{I}}}}}}}_{LP}\,$enables the HP model to learn from the feature similarity between the query data and the feature centers of the previous training data in the metric space.

### Details of baseline performance evaluation

In the MNIST and Fashion-MNIST experiments, we used Bernoulli sampling to encode the pixel into spike trains. In the sequential MNIST and CIFAR10 experiments, we took the first spiking layer as the encoding layer^59^ to convert the pixel information into spike signals. Here, the sequential MNIST is a variant of MNIST dataset, which inputs the original image in a row-by-row pixel manner. In the CIFAR10-DVS and DVS-gesture experiments, we accumulated spike trains (8 and 10 ms, respectively) for acceleration and directly input them into SNNs. We applied the batch normalization (BN) technique to convolutional layers on the DVS-Gesture dataset by following the work^62^. We optimized the HP model in all datasets using the mean square error (MSE) and the adaptive moment estimation optimizer (ADAM). A four-layer MLP with [28-256FC-256FC-10] was trained on sequential MNIST, a six-layer CNN with [input-128C3-AP2-256C3-AP2-256C3-AP2-512FC-10] was trained on MNIST and Fashion-MNIST datasets, a nine-layer CNN with [input-64C3S2-BN-128C3S1-BN-256C3S1-BN-256C3S1-BN-256C3S1-BN-256C3S1-AP2-800 FC-512FC-11FC] was trained on DVS-Gesture datasets, and a nine-layer CNN with the CIFARNet structure^59^ was trained on CIFAR10 and CIFAR10-DVS. We took the local learning rules in Eq. (5) and meta-parameters in Eq. (10) in all experiments unless otherwise stated. In comparison with fine-tuning models, we used the same learning rules and fixed these meta-parameters after random initialization during training. To reduce computation, we equipped the local module in the hidden fully connected layers in all classification tasks. Other parameter configurations can be found in Table 3.

**Table 3:** Parameter settings on different learning tasks.

Parameters	Descriptions	MNIST	FMNIST	CIFAR10	DVS-CIFAR10	DVS-Gesture	Omniglot
Batch size	–	100	100	50	32	12	3
$a$	Gradient width of *H*	0.5	0.5	0.5	0.5	0.5	0.5
${N}_{0}$	Training epochs	100	100	150	150	150	1000
${\tau }_{w}$	Eq. (10)	40 ms	40 ms	40 ms	200 ms	200 ms	300 ms
${k}_{u}$	Eq. (10)	0.6	0.7	0.5	0.3	0.3	0.2
${v}_{th}$	Eq. (10)	0.3	0.4	0.5	0.4	0.4	0.5

### Details of fault-tolerance learning

In the cropping experiment, we increased cropping area gradually on the center of each image or each NVS frame, denoted by ${(2ci)}^{2}$, where *ci* represents for cropping intensity with a range of $0 \sim 14$. In the noise experiment, since salt-and-pepper noise can maintain the spike binary representation, we use it for evaluating the robustness to the noise in the N-MNIST and MNIST experiments. We also increased the proportion of noise region on each image or each NVS frame gradually, denoted by the noise-level (*nl*), where *nl* value refers to the $nl\times 2{e}^{-2}$ region with a range of $0 \sim 14$. All models were pre-trained on the standard training dataset and tested on the cropping (noise) data with the same parameter configuration, network structure [input-512FC-10FC], and the MSE loss. For the distance comparison, we calculated the membrane potential of the first hidden layer in the last timestep as representations to calculate the distance between the incomplete data and the original data. We randomly sampled 1000 testing data from MNIST and plotted its average distance on Fig. 4e, f.

### Details of few-shot learning

The Omniglot is a standard few-shot learning dataset that contains 1623 categories and each category contains 20 samples. In one training episode, we first randomly selected N classes and sampled S sample pairs from each class (called N-way S-shot). Then, we fed the S-labeled samples (named by the presentation time) into the classifier. After that, we randomly sampled a new but unlabeled instance from the same N classes and queried the classifier for the labels. We used four convolutional layers with 3 × 3 kernel size and two strides, followed by a fully connected layer and an N-way classifier layer. We followed the work^31^ to configure the network parameters and divide the training sets and testing sets. During the training phase, we sampled three task episodes from the task distribution $\varGamma$, and used the one-step updated weights ${{{{{\bf{w}}}}}}$ to approximate ${{{{{{\bf{w}}}}}}}^{{{{{{\boldsymbol{\ast }}}}}}}$ using training task samples, and alternatively iterated the meta-parameters ***θ*** by re-sampling from the same tasks. To reduce the computation, we equipped the local module in the fully connected layers. The training label was fed into the last classifier layer by the one-hot coding scheme to guide the correct classifications. We adopted the encoding strategy as used ref. ^59^ to produce spike trains. We trained the network of 3,000,000 episodes and reported the best results over the last 1000 episodes.

### Details of continual learning

The shuffled MNIST experiments include multiple image classification tasks. All tasks are to classify handwritten digits from zero to nine. Each task is a variant of the MNIST dataset with a different permutation. For each new task, the image pixels were randomly permuted with the same randomization across all digits and different randomization are used in different tasks. We trained each task by ten epochs. We adopted a four-layer spiking network with [784-1024-1024-10] structure and minimized the MSE loss by the ADAM. During the training process, we fixed the meta-parameters of the local module and randomly generated a sparse and fixed connection matrix to receive supervision signals. We used the BPTT to update these sparse weights and local learning to update other connections. After each task is learned, we fixed the weights and updated the hyperparameters of the local modules for one epoch. Other comparison methods were adopted from the corresponding publications^25,51,52^ and applied to spiking models.

### Details of hardware implementation

The Tianjic chip is a cross-paradigm neuromorphic computing platform that supports a broad spectrum of neural coding schemes, computational models, and network structures. It is fully digital and fabricated using 28-nm high-performance low-power technology. Each Tianjic chip contains 156 functional cores (FCores), which are arranged in a 2D-mesh manner. Each FCore contains a group of neurons, a group of axons, and synaptic connections between them. Among the FCores, spikes can be transmitted to one or more cores in the mesh through the routing network in a form of routing packets. At the same time, through the inter-chip communication interface, multiple chips can expand the internal routing network connections into a larger computing platform.

We mapped the model onto multiple FCores. Different FCores are configured to perform different basic operations and transformations. Taking the MNIST dataset as an example, we deployed 70 FCores to implement a fully connected structure [784-1024-1024-10]. We tested the energy consumptions with different coding schemes. Because the rank coding shortens the average decision time, it can effectively reduce the on-chip inference latency and the average compute ratio, thereby alleviating average dynamic power consumption. The average on-chip inference latency required for rate coding and rank coding are 0.27 and 0.18 us, the compute ratios are 0.63, 0.45, and the dynamic power consumptions are 0.48 and 0.38 W, respectively. We reported the on-chip performance on MNIST, F-MNIST, and N-MNIST datasets and compared it with GPU-based running results in Supplementary Table 1. With the massive parallelism and the near-memory computing architecture, the execution time on the Tianjic can be much faster than that of the general-purpose computer. The energy consumptions scale only slightly as the network size increases owing to the spike-driven paradigm and local-memory many-core architecture (Fig. 5d).

The cycle-accurate simulator can well capture the hardware chip properties at runtime and is commonly used for chip evaluation. We based on Tianjic’s hybrid structure to design an on-chip hybrid learning scheme and a feasible cycle-accurate hardware simulation scheme to evaluate on-chip computational resources. Here we took an extended version of Tianjic chip with re-configurability and functionalities to support continuous execution of multiple operations (see Supplementary Note 1). On this basis, we developed a mapping scheme to disassemble the overall dataflow into performable fine-grained basic operations and further transformed a mapping design into executable configuration (see Supplementary Note 2). We simulated three on-chip learning modes (LP, GP, and HP) using the software toolchain. A detailed simulation scheme is provided in Supplementary Fig. 1. With this simulation scheme, we estimated the throughput and route cost of different learning modes using an MLP structure [784-512-10] and the time window $T=3$. Regarding the route cost in Fig. 5e, we accumulated the amount of data volume whenever data transmission occurs. Regarding the throughput in Fig. 5f, we recorded the time spent in each phase when executing computational tasks on all allocated FCores. After that, we summed the time consumptions together to count the total clock consumption and thereby the throughput.

## Supplementary information

Supplementary Information

## Data Availability

All data used in this paper are publicly available and can be accessed at http://yann.lecun.com/exdb/mnist/ for the MNIST dataset, https://www.cs.toronto.edu/~kriz/cifar.html for the CIFAR dataset, https://www.garrickorchard.com/datasets/n-mnist for the N-MNIST dataset, and https://github.com/brendenlake/omniglot/ for the Omniglot dataset.

## Code availability

Implementation of hybrid learning model is made public together with the publication of this paper https://github.com/yjwu17/Spiking-hybrid-plasticity-model. Codes related to the detailed hardware implementations are available from the authors upon reasonable request.
