# Supplementary material for: Brain-inspired global-local learning incorporated with neuromorphic computing
Source: Nat Commun. 2022 Jan 10;13:65. doi: 10.1038/s41467-021-27653-2 (PMC8748814; doi:10.1038/s41467-021-27653-2)
Supplement: Supplementary file 1 — Supplementary Information [file 41467_2021_27653_MOESM1_ESM.pdf]

Supplementary Information for: Brain-inspired Global-local Learning Incorporated with Neuromorphic Computing

Yujie Wu[1†], Rong Zhao[1†], Jun Zhu[2†], Feng Chen[3†], Mingkun Xu[1†], Guoqi Li[1], Sen Song[4], Lei Deng[1], Guanrui Wang[1, 5], Hao Zheng[1], Songchen Ma[1], Jing Pei[1], Youhui Zhang[2], Mingguo Zhao[3], and Luping Shi[1*]

[1]Center for Brain-Inspired Computing Research (CBICR), Beijing Innovation Center for Future Chip, Optical Memory National Engineering Research Center, Department of Precision Instrument, Tsinghua University, Beijing, China

[2]Department of Computer Science and Technology, Tsinghua University, Beijing 100084, China

[3]Department of Automation, Tsinghua University, Beijing 100084, China

[4]Laboratory of Brain and Intelligence, Department of Biomedical Engineering, IDG/ McGovern Institute for Brain Research, CBICR, Tsinghua University, Beijing, China

[5]Lynxi Technologies Co., Ltd. Beijing, China

[†]These authors contributed equally to this work.

[*]e-mail: lpshi@mail.tsinghua.edu.cn.

Supplementary Table 1: Performance evaluation of hybrid models implemented on different computational platforms.

Dataset	Platform	Coding	Acc. (%)	Compute ratio	Power (W)	Energy (mJ)	Latency (ms)
MNIST	GPU	Rate	99.55	-	37.13	338.99	9.13
	Tianjic	Rate	99.43	0.46	9.35	3.46	0.37
	Tianjic	Rank	99.22	0.27	9.22	2.56	0.23
F-MNIST	GPU	Rate	93.45	-	39.23	588.24	14.23
	Tianjic	Rate	93.30	0.46	9.56	3.53	0.37
	Tianjic	Rank	93.11	0.33	9.37	2.80	0.30
N-MNIST	GPU	Rate	99.53	-	49.97	1214.8	24.31
	Tianjic	Rate	99.45	0.46	13.31	4.93	0.37
	Tianjic	Rank	99.21	0.28	13.00	3.65	0.28

Supplementary Note 1: Design of three learning methods on Tianjic chips

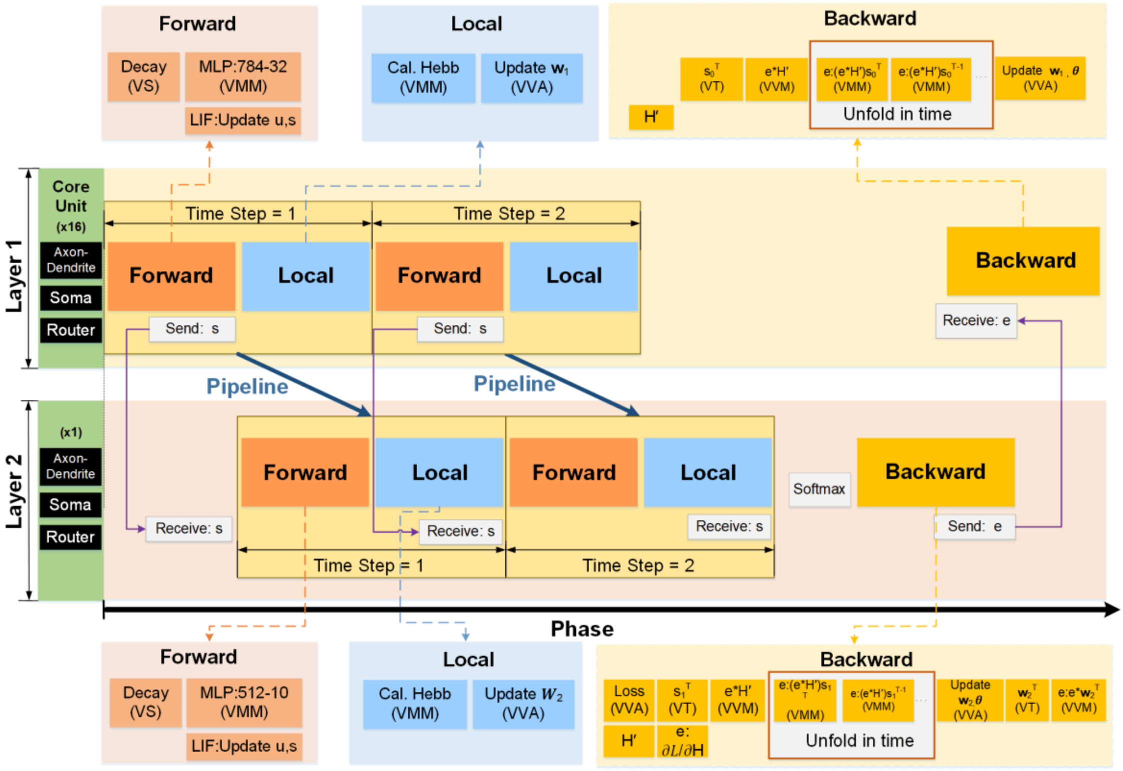

Supplementary Figure 1. Illustration of the proposed hybrid on-chip learning implementation scheme. The left black boxes denote the main units of function cores (Fcores), in which every operation is executed and aligned in a phasic form. The inference process contains two modules: a forward module in jacinth boxes and a local module in light blue, carrying out for T times. The intermediate variable u, s represent the membrane potential and spike firing pattern during the inference procedure, where H denotes the Heaviside function. The following backward process (the orange boxes) calculates error information and propagate in a reverse direction to update weights parameters and meta-parameter $\boldsymbol{\theta}$. And e stands for the temporary error calculated layer-by-layer. The abbreviations in the bracket of Forward, Local and backward boxes are the basic executable operations supported by the Tianjic platform. Please refer to ref.[1] for details. The purple arrows represent cross-layer routing paths. The forward path and local path are performed in a pipelined manner.

We designed a comprehensive on-chip hybrid learning scheme with a configurable software tool chain from algorithm to hardware to emulate the forward and backward paths in three learning modes. Specifically, we developed a dual spatial-temporal unfolding mapping scheme (DST-UM), a mapping compiler for network partition and resource placement, and a cycle-accurate simulator satisfying chip-specific constraints. In this manner, we evaluated the model performance under different workloads,

including single-paradigm and hybrid-paradigm modes. The mapping implementation scheme of hybrid on-chip learning is presented in Supplementary Figure 1.

As shown in Supplementary Figure 1, we disassembled the dataflow of the entire hybrid learning into basic operations which can be supported by the hardware platform. Here, the calculation between input spikes and weight is split into multiple spatial basic operations due to resource limitation, including fan-in and fan-out, memory space in function cores (FCores). In addition, the proposed DST-UM scheme enables the execution data flow to be allocated and optimized in a single spatial dimension or temporal dimension. Specifically, for the spatial dimension, the parameters and the computation of the first layer were allocated into 16 FCores for balancing computation and memory overhead. Meanwhile, the task of the second layer was allocated into another FCore. On this basis, layer-wise data communication and arrangement can be performed across multiple FCores. For the temporal dimension, the DST-UM unfolded the operations of forward and backward passes along with a timing order. As shown in Supplementary Figure 1, the forward path was organized along with time window and the backpropagation path was unfolded according to the derivation compute dependence. By doing so, the operation of the entire process can be mapped onto one chip in a spatially parallel and temporally serial manner. Finally, we organized this procedure according to algorithmic details. In the inference process, we propagated spike signals layer-by-layer and updated local items (the orange boxes in Supplementary Figure 1) in each timestep. After performing the inference for T times, we updated the weights and meta-learning parameters $\boldsymbol{\theta}$ along the backward path (the orange blue boxes in Supplementary Figure 1).

Given the above mapping implementation, we used a software tool chain reported by ref.[1], which includes a mapping compiler used for network partition and resource placement, and a C++-based cycle-accurate simulator used for hardware simulation that considers all chip-specific constraints. We used the compiler to perform placement and generate configured file automatically. Therefore, our simulator can utilize the configuration and simulate the running process to generate the corresponding analysis results and evaluation. We configured each FCore according to Supplementary Figure

1 and thereby used the software tool train to simulate the entire process of hybrid learning. Notably, we also simulated the event-driven attributes and the sparse processing of intermediate variables in three modes. We utilized the parallel processing mechanism of the many-core architecture to carry out all inference process. The LP circuit was performed in a pipelined manner and the backward process of GP and HP were performed in a serial manner. Finally, we based on this scheme and estimated the computational resources of online learning.

Supplementary Note 2: Details of hardware evaluation methods

We chose the three models (LP, GP, and HP) with the same MLP structure [784-512-10] as an example, and set the time window at $T = 3$. We mainly took the following steps during implementation, which are described as follows:

(1) **Designing mapping scheme.** We used the Tianjic mapping scheme to disassemble the overall dataflow into performable fine-grained basic operations. Considering the huge number of parameters in the first layer, we allocated the computation task of the first layer into 16 function cores for balancing the compute and memory overhead. Then, we allocated the second layer into one FCore, where layer-wise data communication and arrangement can be performed;

(2) **Configuring software toolchain.** Based on the mapping design, we further determined the key operations and emulated the process of on-chip learning. All operations are divided into three basic units of FCores, including Axon-Dendrite, Soma and Router. We initialized the network parameters of specific models according to hardware constraints and further transformed the mapping scheme into a specific executable configuration with the Tianjic software simulation toolchain[1]. By doing so, we can simulate the three on-chip learning modes (LP, GP, and HP) using the Tianjic software platform.

(3) **Simulating on-chip running process and data arrangement**. With the proposed mapping scheme, we estimated the entire online learning process of different learning modes using the Tianjic simulation platform. In Tianjic, the entire process is executed via a group of phases in a sequential manner, where each phase contains the operations

from the subset of three basic units (Axon-Dendrite, Soma and Router). Hence, we collected the associated running cost of each operator in each unit, such as memory consumption, communication data volume and running clock cycles. And then we obtained the implementation cost.

To better illustrate the evaluation, we first briefly introduce some basic concepts and definitions and then elaborate on the specific computation methods related to the throughput and route cost.

Mathematical abstract preliminaries of the simulation process. Assume that a task process is performed as a set of phases $\mathbb{C}= \{Phase_1, \ldots, Phase_i, \ldots, Phase_N\}$, wherein N denotes the number of phases required to run a complete cycle. The i_{th} phase $Phase_i$ is an operator set which contains fine-grained functional operations supported by the three basic units. If we denote the operator sets of Axon-Dendrite unit, Soma unit and Router unit by O_{AD}, O_S, O_R, respectively, it yields $Phase_i \subseteq \{O_{AD}, O_S, O_R\}$. Please note that in the throughput cost evaluation, we used O_u to refer to any of the three operator sets, and configured O_u according to the task requirements and mapping schemes to realize the calculation of the three models as shown in Supplementary Figure 1.

Regarding to the route cost evaluation. The route cost (RC) yields the following equation:

$$RC = \sum_{i \in \{v | Phase_v \cap O_R \neq \emptyset\}} CoreN_i \sum_{q}^{Q_i} Packet_q.$$

Here the communication data volume $Packet_q$ is carried by the q_{th} route packet and measured through the simualtion process. Q_i denotes the total number of route packet in the i_{th} phase. $CoreN_i$ denotes the allocated number of FCores performing one task in parallel in the i_{th} phase. $\mathrm{Phase}_i \cap O_R \neq \emptyset$ means that we only record the phases involving with inter-core communication.

Regarding to the throughput cost evaluation. We recorded the time spent in each phase when executing computational tasks on all allocated FCores. The throughput cost (TC) yields the following equation:

$$TC = \frac{F}{\sum_{i=1}^{N} \sum_{\mathrm{u} \in \{v | O_v \in Phase_i\}} \max_{k \in \{1,2,..,CoreN_i\}} Clock_k^u},$$

where F denotes the clock frequency of the simulator, u denotes different operator types from $\{O_{AD}, O_S, O_R\}$, $Clock_k^u$ denotes clock consumption of the k_{th} allocated core in the u_{th} type operator of $Phase_i$. We recorded the maximum number of the u_{th} operator among $CoreN_i$ cores in $Phase_i$ for computation.

We accumulated the data volume whenever data transmission occurs, and summed the time consumptions together to count the total clock cycles. By combining the recorded data and evaluation formulas, we computed the route cost and throughput cost, and obtained the results in Fig.5e and Fig.5f.

Regarding the energy consumption. We pre-trained the HP models with different network sizes and allocated the FCores for the corresponding network sizes. On this basis, we ran the inference process on the Tianjic using the mapping tools and measured the power consumption and running time. Finally, we multiplied them and obtained the energy consumption shown in Fig. 5d.

Supplementary Note 3: Linking the HP SNNs with rank order coding

We first give an overview of rank order coding[2] and then analyze its relationship with the HP model.

Rank order coding assumes that the biological neurons can encode information by firing orders across a neuron population. Assume that the target neuron i receives inputs from a presynaptic neuron population A_l, and each neuron only fires a spike once. Let activations of afferent neurons be a_j^l. Then the rank order coding can record the relative firing orders of afferent neurons, and update the activation of a_i^{l+1} by,

$$a_i^{l+1} = \sum_{j \in A_l} r^{order\left(a_j^l\right)} w_{ij}^{l+1}, \quad (1)$$

where $r \in (0,1)$ is a given punishment constant, $order(a_j^l)$ is the firing order of neuron j in the presynaptic population. Equation (1) shows that the ranking factor $r^{order\left(a_j^l\right)}$ is a key for the rank order coding, which can encourage the early firing of

while punish the later firing of the neuron. Next, we show that the ranking factor can be equivalently converted into the decay function of our model, which indicates that the information propagation of the HP model encodes information by equation (1).

Theorem 1: Assume each neuron fires at most one spike in a given short time window. The HP model encodes information in a form of rank order coding.

Proof: we first formalize the input current I of equation (1) as

$$I = \sum_{j=1}^{l_n} r^{order\left(s_j^l\right)} w_{ij}^{l+1} s_j^l,$$

where we add a spike signal $s_j^l \in \{0,1\}$ to incorporate all presynaptic neurons and make the updating compatible with the above neuron update equations. By formula deformations, it holds

$$I = \sum_{j=1}^{l_n} w_{ij}^{l+1} e^{log(r) order\left(s_j^l\right)} s_j^l = \sum_{j=1}^{l_n} w_{ij}^{l+1} e^{-\frac{order\left(s_j^l\right)}{-\frac{1}{log(r)}}} s_j^l = \sum_{j=1}^{l_n} (w_{ij}^{l+1} e^{-\frac{t_m - t_0}{\tau_w}}) s_j^l,$$

where $\tau_w = \frac{1}{-log(r)}$, $order\left(s_j\right) = t_m - t_0$. In this manner, the ranking factor $r^{order\left(s_j^l\right)}$ can be equivalently converted into the decay function of the HP method.

Supplementary Note 4: Meta-learning spike timing-dependent learning rule

The proposed HP model provides a general method to learn spike-based local plasticity by parametrizing the plasticity function $P\left(t, pre_j(t), post_i(t), w; \boldsymbol{\theta}\right)$ and using the proposed meta-learning method. Here we take the spike timing-dependent learning (STDP) rule as another demonstration.

We used the synaptic trace method[3] to record the pre- and post-neuronal firing activity and established an STDP-based HP model. Specifically, we use a synaptic trace variable x_{pre} to keep the spike history of each presynaptic neuron as follows

$$\tau_s \frac{dx_{pre}}{dt} = -x_{pre} + \sum_{t_f < t} s\left(t - t_f\right),$$

where τ_s denotes the decay constant of synaptic trace. When a spike arrives at this synapse, x_{pre} is increased by 1; otherwise x_{pre} decays exponentially by a decay factor τ_s. Likewise, we use x_{post} to keep the spike history of each postsynaptic neuron. By doing so, the local plasticity variables P can be replaced by

$$P(t_m) = P(t_{m-1})e^{-\frac{dt}{\tau_w}} + A_+ s_{pre} x_{post} - A_- s_{post} x_{pre},$$

where the local hyperparameter, such as A_+ and A_-, can be meta-learned by the proposed method.

Reference

1 Deng, L. *et al.* Tianjic: A unified and scalable chip bridging spike-based and continuous neural computation. *IEEE Journal of Solid-State Circuits* **55**, 2228-2246 (2020).

2 Thorpe, S. & Gautrais, J. in *Computational neuroscience* 113-118 (Springer, 1998).

3 Diehl, P. U. & Cook, M. Unsupervised learning of digit recognition using spike-timing-dependent plasticity. *Frontiers in computational neuroscience* **9**, 99 (2015).
